# FRAILTY AND THE COMPARATIVE EFFECTIVENESS AND SAFETY OF SGLT2I AND SULFONYLUREA IN OLDER ADULTS WITH TYPE 2 DIABETES

**DOI:** 10.1093/geroni/igz038.2156

**Published:** 2019-11-08

**Authors:** Ajinkya Pawar, elisabetta Patorno, Dae Kim

**Affiliations:** 1 Brigham and Women’s hospital, Boston, Massachusetts, United States; 2 Hebrew SeniorLife, Boston, Massachusetts, United States

## Abstract

We conducted a 1:1 propensity score-matched retrospective cohort study of 70,826 patients with type 2 diabetes (mean age, 71.4 years [standard deviation, 5.0]) initiating a SGLT2i or a second-generation sulfonylurea in Medicare data. We estimated HRs (95% CIs) for a composite cardiovascular endpoint and severe hypoglycemia comparing the two treatments in the entire population and by the CFI-based frailty subgroups. Compared with sulfonylureas, SGLT2is were associated with lower rates of the composite cardiovascular endpoint (HR, 0.68 [95% CI, 0.62-0.75]) and severe hypoglycemia (0.43 [0.35-0.53]) over a mean follow-up of 9.5 months. The lower rate of composite cardiovascular endpoint for SGLT2i vs sulfonylureas was observed in pre-frail (0.68 [0.61-0.77]) and frail (0.64 [0.53-0.77]) subgroups, but not in non-frail subgroup (0.95 [0.59-1.54]). The rate of severe hypoglycemia was consistently lower for SGLT2i vs sulfonylureas across frailty subgroups (non-frail, 0.37 [0.12-1.16]; pre-frail, 0.45 [0.35-0.59]; frail, 0.40 [0.28-0.58]).

